# Address of the Retiring President to the West Texas Medical Society

**Published:** 1889-12

**Authors:** P. W. Johns

**Affiliations:** Retiring President


### SOCIETY NOTES.

For Daniel's Texas Medical Journal.

### ADDRESS BEFORE THE WEST TEXAS DISTRICT MEDICAL SOCIETY.

BY THE RETIRING PRESIDENT, DR. P. W. JOHNS.

GENTLEMEN:—To-night for the second time during my ten years residence in Texas, my official term as President of a Medical Association expires. With what ability I have served you, is for you to say. While the pleasure of the honor you have conferred upon me, is for me to enjoy and remember.

Upon vacating this chair to my honored successor, the pleasing duty of addressing you is by the constitution imposed upon me. A privilege and opportunity waived by most of my predecessors, yet one I would gladly grasp had I the ability to interest or enlighten you upon the many themes pregnant with interest to the profession, upon the intelligent solution of which rest not only our professional welfare, but the public weal and the common good.

While there is much in the past year's record of the Association worthy of its congratulation, yet a retrospective look over my administration recalls one or more unfortunate occurrences in the society, while perhaps unavoidable, yet are to me, and I believe to most of you, a source of deep regret, and should be referred to, or remain on the records, only as signals of danger in the future, and I gladly dismiss them forever with the earnest hope that their harmful effects may exist no longer. I am pleased to congratulate you upon the present healthy state of the Association.

All of our meetings have been well attended. There were admitted during the year thirteen new members, and there were three resignations, giving the Association a working membership of forty-one. In the plentitude of His mercy, the great physician, Our Heavenly Father, staid the hand of death from our midst for His own good purpose. For whose mercy let us ever be mindful and invoke His continued blessings and preservation. Yet, within the last few days death has claimed for its own, *one who though with us as a member but a brief season, left upon the records of this society forever the impress of his counsel and genius.

During the past year the society has inaugurated a work contemplated in its original formation; that of extending its membership over Southwest Texas, and thereby, becoming in fact, as well as in name, The West Texas Medical Association, the success of which is evidenced by two encouraging meetings, and by the presence of some of you to-day.

During the past year the members of this society have determined that its by-laws and constitution were not merely idle and stereotoped statutes, but that they were inviolate and must be obeyed.

We can truthfully boast of some of the ablest scientific productions and discussions that have ever been the product of this society.

Not so many we admit, yet because this Association has had

---

* Dr. M. K. Taylor.

its time and attention for a part of the time during the year diverted from scientific investigation does not entitle it to the adverse criticism it has received from some disaffected sources. It evidences neither a lack of inclination nor an inability on the part of its members for scientific work. For while scientific investigation and discussion is the paramount object of medical associations, yet their usefulness and perpetuity rest not alone upon scientific research, but also upon the correction of profession abuses; nurtures and regard for ethical principles; the inspiring of brotherly esteem and social improvement among its members, and a fidelity to its senior organizations, making themselves subsequent to the needs and general wellfare of the profession at large in promoting its causes, and in the execution of its purposes.

This Association is to be congratulated upon the handsome and satisfactory manner with which it received and entertained the State Association in April last. While we received material aid from the municipal government and citizens at large, yet each of you gave with a liberal hand, and it must ever be a source of pride to you and a credit to this society that the entertainment of the State Association as the guests of this society was so liberal and complete as to merit the plaudits of the profession throughout the State. It is meet and proper that the collective branches of the profession, as well as the individual members, be ever ready to lend their moral, financial and professional support to the mother society—the State Association—or else the perpetuity of its usefulness will be endangered. The enormous territory covered by the membership of the State Association, the continued birth and rapid growth of district societies in distant parts of the State, have in the minds of some, already threatened the downfall and dissolution of your State society. God forbid! And I say to you to-night, if we will only not be recreant to our duty, instill a becoming patriotism and proper management into our district societies, these very physical and professional conditions seemingly adverse to the interest of the State Association, will only redound to its good, and tend to cement and strengthen it in its moral course to greater usefulness and grander achievements.

And I speak the sentiments of the West Texas Medical Association when I say, enobled by a maternal love and patriotic pride, by her glorious achievements in the past, and her honorable purposes in the future, she holds dear and guards with a zealous care her steadfast fidelity and allegiance to the State Association, and will ever remain a stone in that monument reared to commemorate the perpetuity of her usefulness—one and indivisible! But let me here sound a note of warning, and God forbid that our professional intelligence should ever be questioned, its honor aspersed, its usefullness impaired, or its pride humiliated, or that it should be dethroned from the exalted position in the estimation of the world. But if I read correctly the signs of the times, the strength, and patronage, the power and influence of the regular profession of medicine among the people is on the wane. And should we fail to stand shoulder to shoulder, county, district, State and nation in the correction of the adverse conditions, the dissolution and downfall of your beloved organization is at least problematical.

A bold assertion and a humiliating confession. But, if it be not so, why demand ye in vain for so long a time a proper recognition in the courts of the country? If it be not so, why rap ye in vain, for so many years, at the door of your capitol for legislative enactments regulating the practice of medicine? If it be not so, why to the commercial departments of the profession come twelve orders for patent nostrums, for every one ℞ that is filed? Why the birth and growth of so many dogmas of the day? If it be not so, why these jeers and criticisms with which we are taunted by the press of the country?—a fair index of public sentiment. And if it be not so, why this success and fortune that follows in the wake of quacks?

The grand potentate of quackery,—the flower of the flock, lounging in his gilded palace car, in his annual triumphant tour over this fair land, stops but a day in your city. His coming has been heralded, and from your own people in twenty-four hours he gathers into his coffers a wealth that might put to shame the bank account of some of us. If you answer, these things are but local, I warn you that among all the civilized nations of the world the United States of America stands alone without *national* laws regulating the practice of medicine. Let us pause but time enough to ask how may we change these conditions and avert the danger?

I answer, organization at home, and fidelity to the cause abroad is the slogan, by which we can bar forever the door to quackery, that to this good hour on the hinges of ignorance stands ajar!

It well behoves us to organize for the advancement and protection of every interest of the profession,—guard its portals by elevating the standard of your schools,—continue to demand the needed legislation for the correction of its abuses and let our own walks insure its purity and maintain its dignity.

Your code should conform to this progressive age. Based upon broad, liberal, progressive, yet dignified principles; demanding no exclusive rights, and recognizing all that is honorable, humane and intelligent.

The sublime sentimentality that clusters about and is inherent to the profession, is a better guide and higher inspiration to its disciples than any written code,—moulding their life to an honest, conscientious performance of duty. Thus organized, armed and inspired in such a cause, there awaits you no failure, but a *destiny*,—would to God I could unfold. A destiny, by the attainment of which you give a blessed heritage to all mankind. To the faithful, conscientious physician belongs the highest reward in this life, and the richest heritage in the life to come.

Glance down the corridors of the past; study well the present; or peer through the mist of the future, and what profession, creed or class do you find in the vanguard of battle for the relief and advancement of their fellow-man? A grateful humanity answers: The profession of medicine.

From the time of Æsculapius to this good hour, the profession of medicine, all along both its dark and its illustrious career, has planted seed of good by the wayside, the abundant fruition of which has been a succor and relief to a suffering humanity. Heroes may boast of their valor and glory in the number of their slain,—they may mingle the shouts of triumph amongst the graves of the dying, and exult over prostrate humanity. They may proudly bear away the laurels of victory from the field of carnage, but the glory of medicine consists not in making wounds, but in binding them up; not in destroying life, but in saving it; and her ptoudest chaplets are the spontaneous offerings of grateful hearts.

